# High Throughput FISH Screening Identifies Small Molecules That Modulate Oncogenic lncRNA MALAT1 via GSK3B and hnRNPs

**DOI:** 10.3390/ncrna9010002

**Published:** 2023-01-03

**Authors:** Nina Zablowsky, Lydia Farack, Sven Rofall, Jan Kramer, Hanna Meyer, Duy Nguyen, Alexander K. C. Ulrich, Benjamin Bader, Patrick Steigemann

**Affiliations:** Nuvisan ICB GmbH, Müllerstrasse 178, 13156 Berlin, Germany

**Keywords:** FISH based high-content screening, novel MALAT1 destabilizer, involvement of GSK3B in MALAT1 regulation

## Abstract

Traditionally, small molecule-based drug discovery has mainly focused on proteins as the drug target. Opening RNA as an additional target space for small molecules offers the possibility to therapeutically modulate disease-driving non-coding RNA targets as well as mRNA of otherwise undruggable protein targets. MALAT1 is a highly conserved long-noncoding RNA whose overexpression correlates with poor overall patient survival in some cancers. We report here a fluorescence in-situ hybridization-based high-content imaging screen to identify small molecules that modulate the oncogenic lncRNA MALAT1 in a cellular setting. From a library of FDA approved drugs and known bioactive molecules, we identified two compounds, including Niclosamide, an FDA-approved drug, that lead to a rapid decrease of MALAT1 nuclear levels with good potency. Mode-of-action studies suggest a novel cellular regulatory pathway that impacts MALAT1 lncRNA nuclear levels by GSK3B activation and the involvement of the RNA modulating family of heterogenous nuclear ribonucleoproteins (hnRNPs). This study is the basis for the identification of novel targets that lead to a reduction of the oncogenic lncRNA MALAT1 in a cancer setting.

## 1. Introduction

A large extent of small molecules in the clinic focus on proteins as drug targets. However, protein coding genes only represent a minority of the transcribed genome. mRNAs and especially lncRNAs (long non-coding RNAs, i.e., transcripts > 200 nucleotides) can contain structured regions that may be exploitable for small molecule drug interactions. Therefore, RNA is viewed as a possible alternative target class for small molecules and RNA-targeting lead finding approaches have been gaining interest in recent years [1,2,3]. Harnessing RNA as a drug target would not only open possibilities for so far undruggable protein targets but would also extend the target space to structural RNA species such as lncRNAs. Indeed, attempts using antisense oligonucleotides or siRNA illustrates the power of this approach [1].

In contrast to proteins, in many cases no clear activity has been assigned to structured regions of lncRNA and it is not clear if the binding to such regions by small molecules will indeed interfere with lncRNA function. Most approaches for identifying novel RNA interacting compounds use cell-free systems that do not take RNA-protein interactions and the cellular context into account and do not monitor the impact of compounds on RNA stability in living cells. Therefore, novel high-throughput compatible approaches to monitor RNA stability in response to small molecule treatments are needed [2,4,5].

MALAT1 (metastasis-associated lung adenocarcinoma transcript 1) is a lncRNA consisting of more than 8000 nucleotides [6] and is located in nuclear speckles, where it regulates splicing and transcription [7]. While MALAT1 is highly conserved among species and widely expressed in normal tissues [8], it is overexpressed in several cancers and its expression correlates with poor overall patient survival [7,9,10]. Accordingly, MALAT1 is considered as a potential anti-cancer drug target. Indeed, several studies have shown the beneficial role of MALAT1 downregulation on cancer or metastasis progression in several types of cancer [7,10]. Additionally, MALAT1 is shown to play a potential role in liver diseases and lung injury [11,12]. Most work on MALAT1 downregulation has concentrated on approaches which use genetic deletion by nucleases or synthetic oligonucleotides to feed MALAT1 to the targeted RNA-degradation machinery [7]. A 3′ highly stable triple helix, formed by the expression and nuclear retention element (ENE) and the A-rich region of MALAT1, is predicted to be responsible for MALAT1′s long half-life time of 9–12 h [13]. This discovery triggered the identification of the first MALAT1 binders against this region [14,15]. Furthermore, besides directly targeting MALAT1, aiming at cellular mechanisms responsible for MALAT1 stabilization could prove to be viable for the induction of MALAT1 downregulation [7].

To identify compounds and pathways that modulate MALAT1 levels in a cellular setting either by direct binding or by indirectly interfering with factors required for MALAT1 stabilization, we screened a library of compounds with known bioactivity.

For this purpose, we established a protocol for small molecule FISH (fluorescence in situ hybridization) screening on 384 W microtiter plates and used a probe set of approximately 20 target-specific oligonucleotide pairs and sequential branched-DNA amplification to visualize MALAT1 RNA with high specificity and single molecule sensitivity [16]. As control for compounds acting via general transcription inhibiton, c-myc mRNA was co-visualized. Using this method, we identified and validated a set of compounds that modify nuclear MALAT1 levels. Mode-of-action studies suggest a novel cellular regulatory pathway that regulates MALAT1 lncRNA stability by GSK3B activation and involvement of heterogenous nuclear ribonucleoproteins (hnRNPs).

## 2. Results

The long non-coding RNA MALAT1 is upregulated in a variety of cancers and can be detected in the nucleus of many cancer cells by fluorescent RNA in situ hybridization [16]. As a potential oncogene, it modulates splicing and transcription processes [7]. We set up a high-content-imaging-based HTS (high-throughput screening)-compatible fluorescence in situ hybridization assay to identify novel small molecules that modulate MALAT1 lncRNA levels in cancer cells. MALAT1 lncRNA’s half-life has been reported to be around 9–12 h [17] and therefore general transcription inhibitors are expected as a potential hit class in screens for compounds leading to a reduction in MALAT1 levels, specifically at longer incubation times. To directly identify and exclude general transcription inhibitors in the primary screen, c-myc mRNA with a short half-life was used as a secondary FISH stain. The mRNA transcript of the proto-oncogene c-myc is expected to be localized in single speckles throughout the cytoplasm [18,19]. 

To our knowledge, no high-content small molecule screening compatible FISH on 384-well microtiter plates has yet been reported. Therefore, we miniaturized existing protocols [16,18,20] to 384-well plates. To co-visualize MALAT1 lncRNA together with c-myc mRNA, we utilized multiplexed fluorescent in situ hybridization using a probe set of approx. 20 target specific oligonucleotide pairs and sequential branched-DNA amplification to visualize RNA with high specificity and single molecule sensitivity [16] in Hela and A549 cells, which have been reported to show detectable levels of MALAT1 [16]. Indeed, as expected, Hela cells show strong nuclear staining in a speckled pattern for MALAT1 lncRNA and cytoplasmic speckles for c-myc mRNA, while both RNA species showed similar localization but lower levels in A549 cells (Supplemental Figure S1). Based on higher experession levels, we chose Hela cells for co-FISH of MALAT1 lncRNA and c-myc mRNA in further experiments. As the main readout for compound activity, the median intensity of MALAT1 nuclear stain as well as number of c-myc cytoplasmic speckles was used (Supplemental Figure S1). 

**Figure 1:**
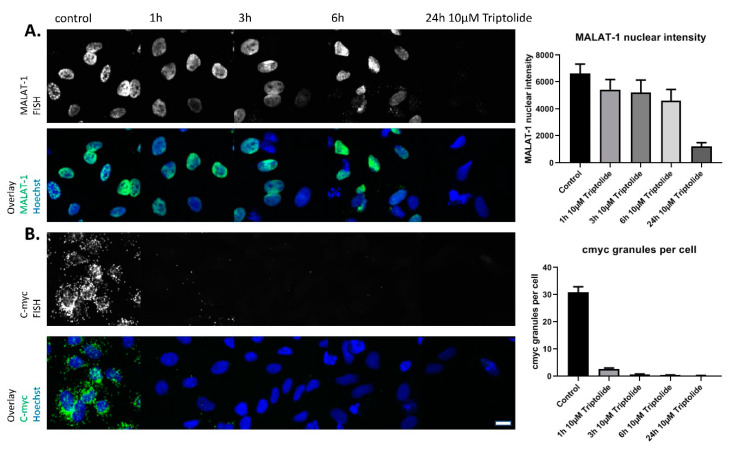
(**A**): Hela stained for MALAT1 lncRNA by fluorescence in-situ hybridization after treatment with transcription inhibitor Triptolide various times. Nuclei are stained by Hoechst. (**B**): Hela stained for c-myc mRNA by fluorescence in-situ hybridization after treatment with transcription inhibitor Triptolide various times. Nuclei are stained by Hoechst. Quantification of nuclear MALAT1 staining or c-myc granules per cell shown on the right. Bars show mean with SD. Scale bar ~10 µm.

Next we tested the decline of the MALAT1 lncRNA nuclear levels as well as c-myc mRNA cytoplasmic speckles after treatment with the transcription inhibitor Triptolide [21]. According to the different reported half-lives of MALAT1 and c-myc, 10 µM Triptolide leads to a fast and complete elimination of c-myc cytoplasmic granules after 1–3 h incubation but showed relatively stable levels of MALAT1 even after 6 h of incubation, with a strong reduction only observed after 24 h of incubation (Figure 1A,B). Therefore, the compound incubation time for the HTS was set to 6 h to allow for sufficient time to develop effects on MALAT1 levels, while c-myc was used as a control to directly exclude general transcription inhibitors and other unspecific hits based on their fast and strong effects as seen on c-myc cytoplasmic levels. 

Based on this, we screened a library of 1664 known bioactive compounds with *n* = 4 at 10 µM (screen concentration and *n* varies for a small part of the compound collection to 10–100 µM) on 24 384 W microtiter plates with DMSO and 1 µM Triptolide as controls (Supplemental Figure S2). Imaging was performed on an automated confocal imaging system using a 20× water objective and with four sites per well located around the middle of the well, resulting in 36 min imaging time per 384-well plate. Screening was robust over the plate set with a mean S/B (signal to background) of 5.9 and a mean RZ’ (robust Z’ factor) of 0.78 and low inter-plate variability (c-myc granules per cell, DMSO vs. Triptolide controls). A dataset comprising the results for all compounds on MALAT1 nuclear levels and c-myc cytoplasmic speckles can be found in the Supplemental Information.

**Figure 2:**
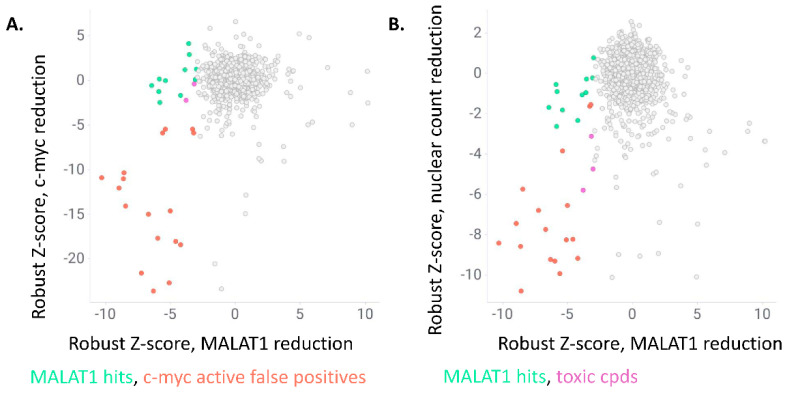
(**A**): Scatter blot showing robust Z-scores for MALAT1 nuclear staining intensity reduction on the *x*-axis and c-myc granule per cell count reduction on the *y*-axis. Compounds with a robust Z-score below −3 for MALAT1 and above −3 for c-myc are considered as hits (in green), compounds with robust Z-scores below −3 for c-myc granule per cell reduction were considered as false positive hits (red). (**B**): Scatter blot showing robust Z-scores for MALAT1 nuclear staining intensity reduction on the *x*-axis and reduction in nuclear counts (i.e., number of viable cells) on the *y*-axis. Compounds with robust Z-scores below −3 for nuclear counts were considered as toxic hits (purple).

In total, we found 31 compounds that reduced MALAT1 nuclear levels with a robust Z-score below −3. Seventeen of these also reduced c-myc cytoplasmic speckles with a robust Z-score of −3 (Figure 2A, marked in red). Indeed, these compounds comprise known general transcription inhibitors (e.g., Actinomycin D and DNA intercalators) and were therefore excluded from the hitlist. Three of the remaining 14 compounds showed a significant reduction in counted nuclei per well and were therefore also excluded from the hitlist as they were potentially driven by cytotoxicity (Figure 2B, marked in purple). 

In total, 11 compounds showed a significant reduction in MALAT1 nuclear intensity (median robust Z-score < −3) without displaying strong effects on c-myc cytoplasmic speckle reduction or nuclear numbers. Interestingly, several hits that are known for interfering with protein homeostasis led to an increase in MALAT1 nuclear intensity without affecting c-myc cytoplasmic speckles (e.g., proteasome inhibitors MG-132 and Bortezomib), however these were not followed in the present study (please see supplement  for full HTS data table). 

**Figure 3:**
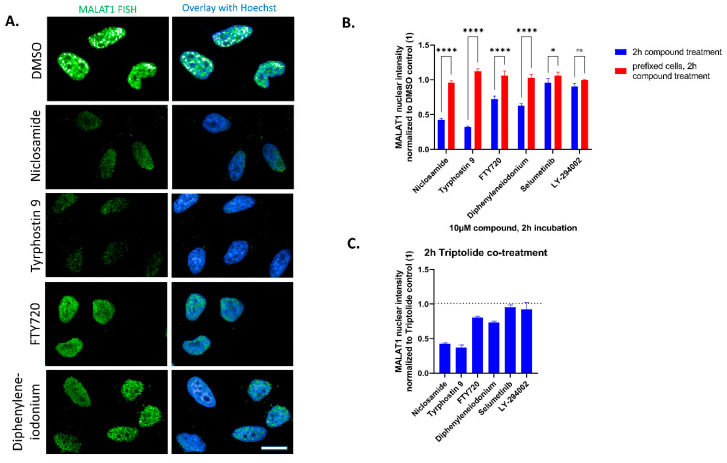
(**A**): Hela stained for MALAT1 lncRNA by fluorescence in-situ hybridization after treatment with DMSO control or HTS hits at 10 µM and 2 h incubation time. Nuclei are stained by Hoechst. Scale bar ~10 µm. (**B**): Quantification of nuclear MALAT1 staining compared with pre-fixed cells after 2 h compound addition. Highly significant effects compared to each pre-fixed control are detected for four of the compounds. Bars show mean with SD. **** *p* value < 0.0001; * *p* value < 0.1; ns = not significant. (**C**): Quantification of nuclear MALAT1 staining after co-treatment with 10 µM of the transcription inhibitor Triptolide and 10 µM of HTS hits. Data normalized to Triptolide/DMSO control (1). Bars show mean with SD.

Six potential hit compounds were reordered and retested at 10 µM and 2 h incubation to validate hits and identify compounds that showed a rapid response on MALAT1 stability. As a control, and to exclude possible false–positive imaging artifacts (e.g., by autofluorescence or quenching or the compound interfering with the probes or detection system), the same compounds were tested on pre-fixed cells (i.e., cells fixed with formaldehyde before compound addition). Each experiment was repeated three times with similar results. In every experiment each condition was tested in three independent wells and with >300 single cells scored per well. Representative results from one experiment are shown in Figure 3. Of the six tested hits, four showed highly significant effects on nuclear MALAT1 levels at 10 µM and 2 h incubation compared with showing no effects on the pre-fixed cells (Figure 3A,B and Supplemental Figure S3).

The identified compounds do not act by general interference with RNA expression as none of the compounds show strong effects on c-myc speckles in the cytoplasm (Figure 2A and Table 1). To strengthen this notion, we tested all compounds in the background of the general transcription inhibitor Triptolide and found that two of the compounds, Niclosamide and Tyrphostin 9, repeatedly maintained a strong reduction in MALAT1 nuclear intensity, confirming the compound effects being independent of transcriptional regulation of MALAT1 (Figure 3C). 

**Figure 4:**
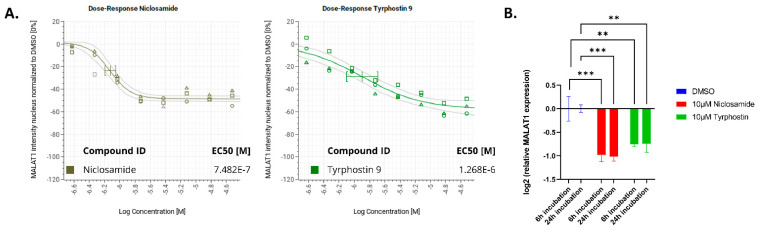
(**A**): EC50 determination of Niclosamide and Tyrphostin 9 for nuclear MALAT1 staining reduction after 2h incubation. Data normalized to DMSO control (0). (**B**): MALAT1 RNA expression levels relative to DMSO control. GAPDH was used as normalization control. Mean +/− SEM (n = 6). *** *p* value < 0.001; ** *p* value < 0.01.

To evaluate compound potency, both hits were tested in dose-response (DR) starting at 30 µM and the EC50 for MALAT1 nuclear reduction was determined (three independent DR curves with n = 3 per concentration each, 2 h incubation (Table 1), being 851 nM +/− 193 nM for Niclosamide and 2.16 µM +/− 1.1 µM for Tyrphostin 9). The maximum efficacy of the compounds plateaued at ~50% reduction for MALAT1 nuclear intensity compared with DMSO controls (Figure 4A). 

To confirm these effects by an orthogonal method, we determined MALAT1 levels by qRT-PCR after Niclosamide or Tyrphostin treatment. Indeed, similar to the effects seen by FISH, incubation with 10 µM Niclosamide or Tyrphostin led to a ~40–50% reduction in total MALAT1 levels compared with DMSO control (Figure 4B).

**Figure 5:**
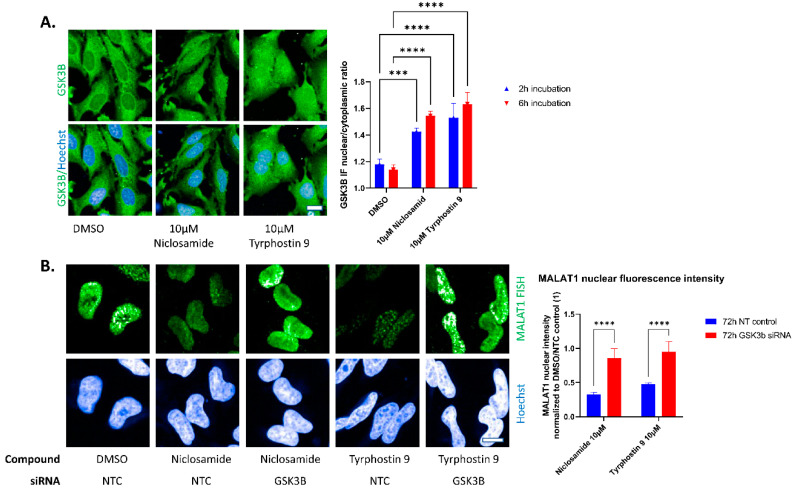
(**A**): Niclosamide and Tyrphostin 9 lead to nuclear translocation of GSK3B. Example pictures (6 h incubation) and quantification of GSK3B nuclear to cytoplasmic levels. Bars show mean with SD. *** *p* value < 0.001, **** *p* value < 0.0001, nuclei are stained by Hoechst, IF against GSK3B. One results from two independent experiments with similar outcomes shown. (**B**): Hela stained for MALAT1 lncRNA by fluorescence in-situ hybridization after 72 h siRNA against the indicated target followed by 2 h treatment with DMSO control or HTS hits at 10 µM. Nuclei are stained by Hoechst. Quantification of nuclear MALAT1 staining after siRNA and compound treatment. GSK3B knockdown significantly prevents compound induced reduction of nuclear MALAT1 staining intensity. Bars show mean with SD. **** *p* value < 0.0001. Scale bars ~10 µm.

No hints for the direct binding of Niclosamide or Tyrphostin 9 to the isolated MALAT1 ENE—A-rich region could be detected by microscale thermophoresis or surface plasmon resonance (Supplemental Figure S4). Therefore, we speculated that the identified hits could act indirectly by modulation of cellular mechanisms that interfere with MALAT1 stability. Niclosamide, as well as several other hits from the hitlist, are reported GSK3B activators [22,23,24,25,26]. Indeed, by immunofluorescence staining against GSK3B, we found a significant relocalization of cytoplasmic GSK3B to the nucleus after treatment with either 10µM Niclosamide or 10 µM Tyrphostin 9 (Figure 5A, antibody validated by siRNA in Supplemental Figure S5A).

Therefore, we tested a possible involvement of GSK3B in the induction of MALAT1 destabilization. For this, we knocked down GSK3B by siRNA for 72 h and measured the ability of Niclosamide or Tyrphostin 9 to reduce nuclear MALAT1 levels. GSK3B siRNA efficiently reduced GSK3B levels (Supplemental Figure S5A) and had no effect on cell viability and significantly prevented reduction in MALAT1 nuclear FISH levels after 2 h treatment with either 10 µM Niclosamide or Tyrphostin 9 (Figure 5B). Similar results were obtained with an alternative siRNA targeting GSK3B (Supplemental Figures S5A and S6). This establishes a role for GSK3B in the modulation of nuclear MALAT1 levels.

GSK3B is a Ser/Thr protein kinase that regulates a myriad of downstream factors and oncogenic events [27]. A literature search identified several GSK3B targets with possible links to MALAT1. While β-catenin is one of the major GSK3B targets, CREB has been shown to potentially directly interact with MALAT1 [28]. 

On the other hand, GSK3B interacts with and regulates the activity of members of RNA binding proteins of the hnRNP (heterologous nuclear ribonucleoprotein) family [27,29,30,31] which in turn have been shown to bind lncNRA and mRNA and regulate RNA stability [31,32]. Indeed, AUF1/hnRNP D, as well as hnRNP A2/B1, C, G, H and hnRNP K, show a direct interaction with MALAT1 [32,33,34,35,36,37,38].

**Figure 6:**
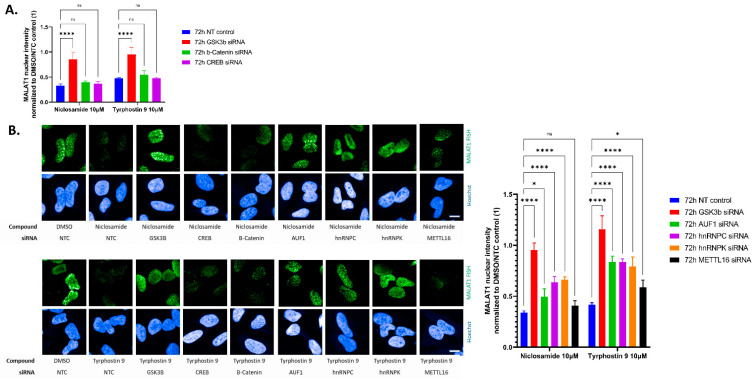
(**A**): Quantification of nuclear MALAT1 staining after siRNA and compound treatment. GSK3B but not b-Catenin or CREB knockdown significantly prevents compound induced reduction of nuclear MALAT1 staining intensity. Bars show mean with SD. (**B**): Hela stained for MALAT1 lncRNA by fluorescence in-situ hybridization after 72 h siRNA against the indicated target followed by 2 h treatment with DMSO control or HTS hits at 10 µM. Nuclei are stained by Hoechst. Scale bar ~10 µm. Quantification of nuclear MALAT1 staining after siRNA and compound treatment. GSK3B as well as to a lower extent hnRNPC and hnRNPK knockdown significantly prevent compound induced reduction of nuclear MALAT1 staining intensity. Bars show mean with SD. **** *p* value < 0.0001; * *p* value < 0.1; ns = not significant.

To identify factors downstream of GSK3B that could be involved in the modulation of nuclear MALAT1 levels, we evaluated if the knockdown of selected GSK3B downstream factors (β-catenin, CREB, selected hnRNPs) prevent the Niclosamide or Tyrphostin 9 induced reduction of nuclear MALAT1 levels. Generally, siRNA led to a robust reduction of the respective target (Supplemental Figure S5B) and, except for the PLK1 siRNA control, siRNA treatment was non-toxic and did not lead to significant alterations in cellular morphology (Figure 6B). Each experiment was repeated three times with similar results. In every experiment each condition was tested in three independent wells and with >300 single cells scored per well. Representative results from one experiment are shown in Figure 6. While we found a strong and highly significant rescue of nuclear MALAT1 levels after Niclosamide or Tyrphostin 9 treatment by GSK3B knockdown in all experiments, neither siRNA against β-catenin nor against CREB prevented the Niclosamide or Tyrphostin 9 mediated reduction of nuclear MALAT1 levels (Figure 6A,B). Interestingly, in comparison to GSK3B knockdown, we found a partial but highly significant prevention of Niclosamide or Tyrphostin 9 induced reduction of nuclear MALAT1 levels after the knockdown of hnRNPC and hnRNPK and partially significant effects for the knockdown of AUF1 (Figure 6B). 

hnRNPs have been reported to require N6-methyladenosine (m6A) RNA modification to enable MALAT1 binding [13,35,36], however, we found no strong effect of METTL16 knockdown, a m6A writer known to interact with MALAT1 [39], on MALAT1 nuclear levels (Figure 6B).

Taken together, the data presented indicates that Niclosamide and Tyrphostin potentially act via the activation of GSK3B and the RNA binding proteins hnRNP K and C, and to a lesser extent AUF1, to modulate nuclear lncRNA MALAT1 levels.

**Figure 7:**
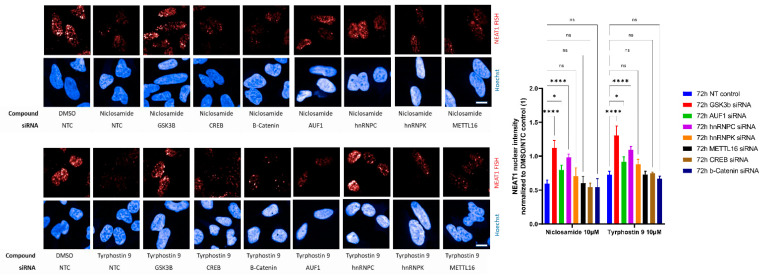
Hela stained for NEAT1 lncRNA by fluorescence in-situ hybridization after 72 h siRNA against the indicated target followed by 2 h treatment with DMSO control or HTS hits at 10 µM. Nuclei are stained by Hoechst. Scale bar ~10 µm. Quantification of nuclear NEAT1 staining after siRNA and compound treatment. GSK3B and hnRNPC knockdown significantly prevent compound induced reduction of nuclear NEAT1 staining intensity. Bars show mean with SD. **** *p* value < 0.0001; * *p* value < 0.1; ns = not significant.

hnRNPs are known to regulate global RNA levels, and therefore the observed effects are not likely specific for MALAT1. Indeed, we find similar effects of Niclosamide on the related lncRNA NEAT1, as well as a rescue of the effects by GSK3B knockdown (Figure 7). However, in contrast to MALAT1, only hnRNPC, but not AUF1 or hnRNPK, shows significant effects on the rescuing of NEAT1 levels after compound treatment (Figure 7), indicating that while GSK3B affect different lncRNAs, fine-tuning on the level of hnRNPs seems to be more lncRNA specific.

**Figure 8:**
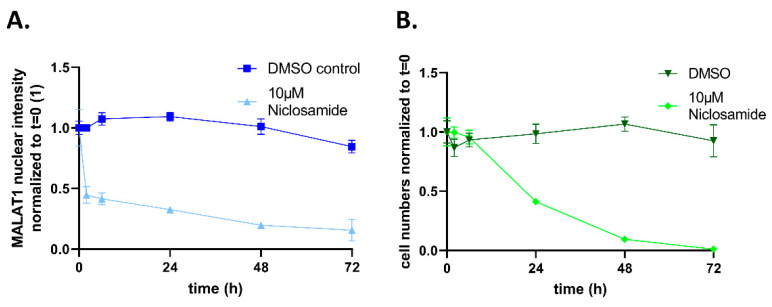
(**A**): Time course of MALAT1 nuclear intensity decrease and (**B**). effect on nuclear numbers. Hela cells were treated with DMSO or 10 µM Niclosamide and MALAT1 was stained by FISH. Nuclear MALAT1 levels and number of nuclei per well were determined. Mean of n = 4 per condition and timepoint with SD as error bars.

Niclosamide is being evaluated as an anti-cancer drug (ClinicalTrials.gov; accessed on 18 December 2022). To evaluate cellular effects after prolonged treatment, we incubated Hela cells with either 10µM Niclosamide or the DMSO control and, by FISH, found a fast and sustained reduction of nuclear MALAT1 levels over a time-course of 72 h. Importantly, this was accompanied by a gradual reduction of cell counts, indicating the effectiveness of Niclosamide as an anti-cancer agent (Figure 8).

## 3. Discussion

MALAT1 is a highly structured oncogenic lncRNA that contains a druggable triple helix structure on its 3′ end required for its stability [15,40,41]. Furthermore, secondary modification by m6A in this region can modulate the accessibility of RNA destabilizing proteins. Specifically, m6A modification seems to alter the local MALAT1 RNA structure to enhance accessibility for the heterogenous nuclear ribonucleoprotein (hnRNP) C [35]. Accordingly, MALAT1 is a potentially suitable target for small molecules, either by the direct binding of the structured regions in the lncRNA or by modification of regulatory elements.

To identify novel compounds that modulate nuclear levels of the oncogenic lncRNA in a cellular setting, we performed what was, to our knowledge, the first reported fluorescence in-situ hybridization (FISH)-based high-content small molecule screen on 384-well microtiter plates and identified several potential GSK3B activators as hits. The involvement of GSK3B in the regulation of MALAT1 stability was validated by knockdown. Several GSK3B downstream factors are potentially linked to MALAT1. Proteasome inhibitors led to MALAT1 accumulation which pointed us to a possible role of β-catenin, whose stability and proteasomal degradation is regulated by GSK3B activity. On the other hand, the transcription factor CREB is regulated by GSK3B and has been shown to directly interact with MALAT1 in the nucleus [28]. However, neither co-addition of a proteasome inhibitor (data not shown) nor β-catenin or CREB knockdown by siRNA were able to rescue the effects of Niclosamide or Tyrphostin 9 on nuclear MALAT1 levels. Instead, we found, through a focused siRNA screening approach, the heterologous nuclear ribonucleoproteins hnRNPC and hnRNPK as the first set of potential downstream targets from the hnRNP family involved in the regulation of nuclear MALAT1 levels. Knockdown of the hnRNP AUF1, which has been reported to directly bind to MALAT1 and NEAT1 and to modulate NEAT1 stability [32], had weaker effects in this setting. Knockdown of hnRNPC and hnRNPK, which have been associated with MALAT1 binding [35,37,38], both show, in comparison with GSK3B knockdown, partial effects on rescuing MALAT1 levels after Niclosamide or Tyrphostin 9 treatment. This indicates that MALAT1 levels are potentially regulated by multiple hnRNPs. On the other hand, NEAT1 levels are sensitive to hnRNPC knockdown, an association that has not yet been reported, but not to hnRNPK and AUF1 knockdown, which indicates that different hnRNPs act on different lncRNAs. 

While the role of GSK3B in the general regulation of mRNA processing is known [42], we demonstrate for the first time the role of GSK3B in the regulation of the stability of the oncogenic lncRNAs MALAT1 and NEAT1. 

By nuclear translocation, GSK3B could potentially directly regulate hnRNPs, such as hnRNPC and hnRNPK, to destabilize nuclear lncRNAs [31,32]. Indeed, a direct interaction between hnRNPC and hnRNPK and MALAT1 has previously been shown [37,38].

It will be interesting to decipher how exactly GSK3B and hnRNPs interact to modulate nuclear lncRNAs. One possible mechanism could be that these factors are involved in the regulation of lncRNA nuclear retention [37,38,43,44]. However, we found no increase in cytoplasmic MALAT1 staining upon compound treatment (data not shown) and reduction of total RNA levels by pRT-PCR. hnRNPs could therefore act to directly destabilize lncRNAs [32,35]. 

As hnRNP are a large family of proteins with partially overlapping functions, this could explain the only partial rescue by the hnRNPs, in contrast to GSK3B knockdown, as well as the differential effects seen on the lncRNAs MALAT1 and NEAT1. N6-methyladenosine modification of lncRNA seems to facilitate lncRNA-protein interactions and GSK3B activity seems to play a direct role in m6A RNA modifications [45]. Furthermore, METTL16 has been reported as binding to MALAT1 [39]. However, initial experiments showed no effect of METTL16 knockdown on MALAT1 levels after Niclosamide or Tyrphostin 9 treatment. Nevertheless, the family of N6m writers is large [46] and their activity on RNA is potentially redundant. Indeed, METTL3 and METTL14 are good candidates for further investigation as N6M writers for MALAT1 [35]. Therefore, it will be interesting to decipher the exact roles for all members of the hnRNP family as well as the N6M writers for different lncRNAs in combinatorial siRNA screens. 

In general, MALAT1 is considered as an oncogene in some cancer settings [47] and therefore small molecules that reduce MALAT1 could be interesting for anti-cancer therapies. We here show that GSK3B activators, such as the oral FDA-approved antihelminthic Niclosamide, could be used to lower oncogenic MALAT1 levels. 

Indeed, Niclosamide is currently under investigation for the treatment of cancer in several clinical trials (ClinicalTrials.gov; accessed on 18 December 2022). However, Niclosamide has a broad range of functions [48] and GSK3B is a central metabolic regulator, being a suboptimal target for compounds with selective activity against MALAT1. Therefore, both have the high potential for unwanted off-target effects in a systemic therapy setting. Indeed, it will be interesting to uncover the range of the transcriptome regulated in response to Niclosamide or Tyrphostin 9 by deep sequencing. Therefore, the identification of the pathways downstream of GSK3B, such as the potential role and interplay of different hnRNP family members and N6A writers, will give rise to more targeted therapy options to reduce nuclear MALAT1 levels.

## 4. Materials and Methods

### 4.1. Cell Culture

Hela and A549 cell lines were obtained from tebu-bio or biomol. Both cell lines were cultured in RPMI1640 Medium (Gibco #21875-034 with 10% FBS (Biochrom #S0615) and 1% Penicillin–Streptomycin (Sigma Aldrich #P0781). Cells were maintained at 37 °C in a 5% CO_2_ and 95% air incubator. For passaging and seeding, cells were washed with DPBS (Gibco #14190-094) and trypsinized (TrypleE Gibco #12604-013). For fluorescence in situ hybridization experiments, cells were plated on 384W microtiter plates (Perkin Elmer Cell Carrier Ultra #6057308). Cells were fixed and permeabilized using ViewRNA Cell Plus Fixation/Permeabilization Solution for 30 min at room temperature, washed once with PBS containing RNase inhibitor and RNA was fixed with RNA-Fixation solution for 1 h at room temperature, followed by washing with PBS containing RNAase inhibitor and optional storage over night in the fridge, all according to the manufacturer’s protocol with adapted volumes for 384 W plates (20 µL/well for washing steps, 15 µL/well for Incubation steps). For time course experiments, cells for all time points were plated together and successively treated with 10 µM Niclosamide at a given timepoint before fixation and FISH staining.

### 4.2. Fluorescence-In-Situ-Hybridization (FISH)

A file containing information according to MISFISHIE [49] can be found in the Supplemental Materials. For FISH, a protocol following the ViewRNATM Cell Plus Assay Kit (Thermo Fisher Scientific #88-19000-99) with probes against MALAT1 and c-myc or NEAT1 (ViewRNATM Probe Sets (MALAT1: VA4-10912; c-myc: VA1-6000107; NEAT1: VA6-14476)) was used. Fixed and permeabilized cells were hybridized in ViewRNA Cell Plus probe solution containing probes against MALAT1 and c-myc or MALAT1 and NEAT1 for 2 h at 40 C, followed by two wash steps with ViewRNA Cell Plus RNA wash buffer. For signal amplification, the ViewRNA cell plus pre-amplifier solution was added and incubated for 1 h at 40 C, followed by two wash steps using ViewRNA Cell Plus RNA wash buffer and incubation for 1h at 40 C with ViewRNA cell plus amplifier solution. After two wash steps with ViewRNA Cell Plus RNA wash buffer, ViewRNA Cell Plus label probe mix was added and incubated for 1 h at 40 C. Cells were washed twice with ViewRNA Cell Plus RNA wash buffer followed by incubation with Hoechst (Biotium #40046, 10 mg/mL; 1:5000) in PBS for 30 min at room temperature. After washing with PBS, PBS was added and the plate was imaged on an Opera Phenix confocal imaging system.

For quantification, MetaXpress or Harmony software were used. Generally, nuclei were identified by Hoechst staining and mean nuclear intensity per cell or granules per cell were determined.

### 4.3. Immunofluorescence

After fixation with ice-cold Methanol, cells were blocked with 1% BSA for 1 h. Mouse anti-human GSK3B (Invitrogen MA5-15597) was used as a primary antibody at 1:500 dilution and after over night incubation and washing, an appropriate secondary antibody conjugated with Alexa Fluor 488 (Jackson ImmunoResearch) was used. Cell nuclei were stained with Hoechst (Life Technologies). Images were acquired on an Phenix confocal spinning disc microscope system (PerkinElmer, Waltham, MA, USA) with a 20× or 40× water objective. Quantification was carried out using Harmony (PerkinElmer, Waltham, MA, USA) and MetaXpress software (Molecular Devices).

### 4.4. qRT-PCR

Total RNA was isolated from cells using RNeasy Plus Mini Kit (Qiagen, Hilden, Germany) and reverse-transcribed with the RevertAid H Minus First Strand cDNA Synthesis Kit (Thermo Fisher, Waltham, MA, USA) according to the manufacturer’s instructions. To measure the expression levels of target genes, sample concentrations were adjusted to 5 ng/µL cDNA and mixed with specific TaqMan Gene Expression Primer (Thermo Fisher, Waltham, MA, USA) and TaqMan Fast Advanced Master Mix (Thermo Fisher, Waltham, MA, USA). Real-time quantification was performed in triplicates on a MicroAmp optical 384-well reaction plate (ThermoFisher) using a QuantStudio 7 Flex System (ThermoFisher). Relative mRNA levels were calculated to the mean of reference gene GAPDH.

### 4.5. Imaging and Image and Data Analysis

Images were acquired using the automated confocal microscopy system Opera Phenix (Perkin Elmer, #HH14001000) and either 20× or 40× water objectives. Image analysis and quantification was carried out with the Harmony (PerkinElmer, Waltham, MA, USA) or MetaXpress software (Molecular Devices) using custom-written scripts. Briefly, nuclei and cytoplasm were detected by Hoechst staining, the number of nuclei were counted per well and the intensity of the FISH signals was quantified as a mean in the region of the nuclei and cytoplasm. For c-myc quantification, c-myc granules were detected as granules per cell. Data was analyzed in Genedata Screener (Genedata, Basel, Switzerland) or Prism (Graphpad, San Diego, CA, USA).

### 4.6. High Throughput Screen

FDA approved and bioactive compound collections comprising 1664 compounds (Enzo FDA approved drug library; #BML-2843 and Enzo known bioactive library; #BML-2840; mainly at 10 mM in DMSO) were transferred to 384W daughter plates with n = 4 wells per compound. Using a Hummingwell (Analytik Jena), 50nl of compound was transferred to a 384 W screening compound plate (Greiner Bio One, #784075) and stored at -20 C. Hela cells were plated as 2000 cells per 384 W in 20 µL medium using a Muldtidrop Combi (Thermo Fisher) and incubated over night. On the day of the screen, screening compound plates were thawed and diluted in 25 µL full cell culture medium by use of a Multidrop Combi (Thermo Fisher) and 20 µL were transferred by CyBio-Well (Analytik Jena) to the cell containing plates and incubated for 6h, after which cells were fixed, permeabilized and stained by FISH (see above). Imaging was performed on an automated Opera System using a 20× water objective and imaging of 4 sites per well located around the middle of the well. Data was analyzed in Genedata Screener (Genedata AG). For robust Z-score computation, the median of the measured signal values of the neutral control (DMSO treated cells) on a plate was subtracted from the measured raw value of a well and divided by the robust standard deviation of the measured signal values of the neutral control (DMSO) wells on a plate.

### 4.7. siRNA Transfection

For silencing, the following oligonucleotides were used (all Horizon/Dharmacon OnTargetPlus siRNA): bCatenin: #L-003482-00-0005; GSK3B: #L-003010-00-0005; CREB: #L-003619-00-0005; HNRNPD: #L-004079-00-0005; HNRNPK: #L-011692-00-0005; HNRNPC: #L-011869-03-0005; METTL16: #L-016359-02-0005; non-targeting control: #D-001810-10-05. Alternative GSK3B siRNA: Ambion Silencer Select pre-designed siRNA against GSK3B (siRNA ID: s6241). The siRNAs were transfected by reverse transfection at a final concentration of 10nM using RNAiMAX (Invitrogen #13778-150) according to manufacturer’s protocol

## Figures and Tables

**Table 1:** HTS hits.

Compound ID	Robust Z-Score: MALAT1 Nuclear Intensity	Robust Z-Score: c-myc Granules per Cell	Robust Z-Score: Nuclear Count	Retest @ 10 µM and 2 h	EC50 Determination
Niclosamide	−5.906422	0.1783263	−2.623441	Strongly active; >50% reduction	851 nM +/− 193 nM
Tyrphostin 9	−3.908122	1.203798	−1.069688	Strongly active; >50% reduction	2.16 µM +/− 1.1 µM
FTY720	−5.875497	−2.437235	−0.8817972	active	
Diphenyleneiodonium	−4.243739	−1.65379	−2.316559	active	
Selumetinib (AZD6244)	−6.467096	−0.541567	−1.678847	inactive	
LY-294002	−3.060566	1.285709	0.7897846	inactive	

## Data Availability

Primary HTS data can be found in the Supplement.

## Supplementary Materials

The following supporting information can be downloaded at: https://www.mdpi.com/article/10.3390/ncrna9010002/s1, information on FISH according to MISFISHIE, raw HTS data table, Supplementary Material and Methods.
