# Supplementary material for: High Throughput FISH Screening Identifies Small Molecules That Modulate Oncogenic lncRNA MALAT1 via GSK3B and hnRNPs
Source: Noncoding RNA. 2023 Jan 3;9(1):2. doi: 10.3390/ncrna9010002 (PMC9844399; doi:10.3390/ncrna9010002)
Supplement: Supplementary file 1 [file ncrna-09-00002-s001.zip › 221207_Zablowsky_supplemental_Figures.pptx]

## Slide 1
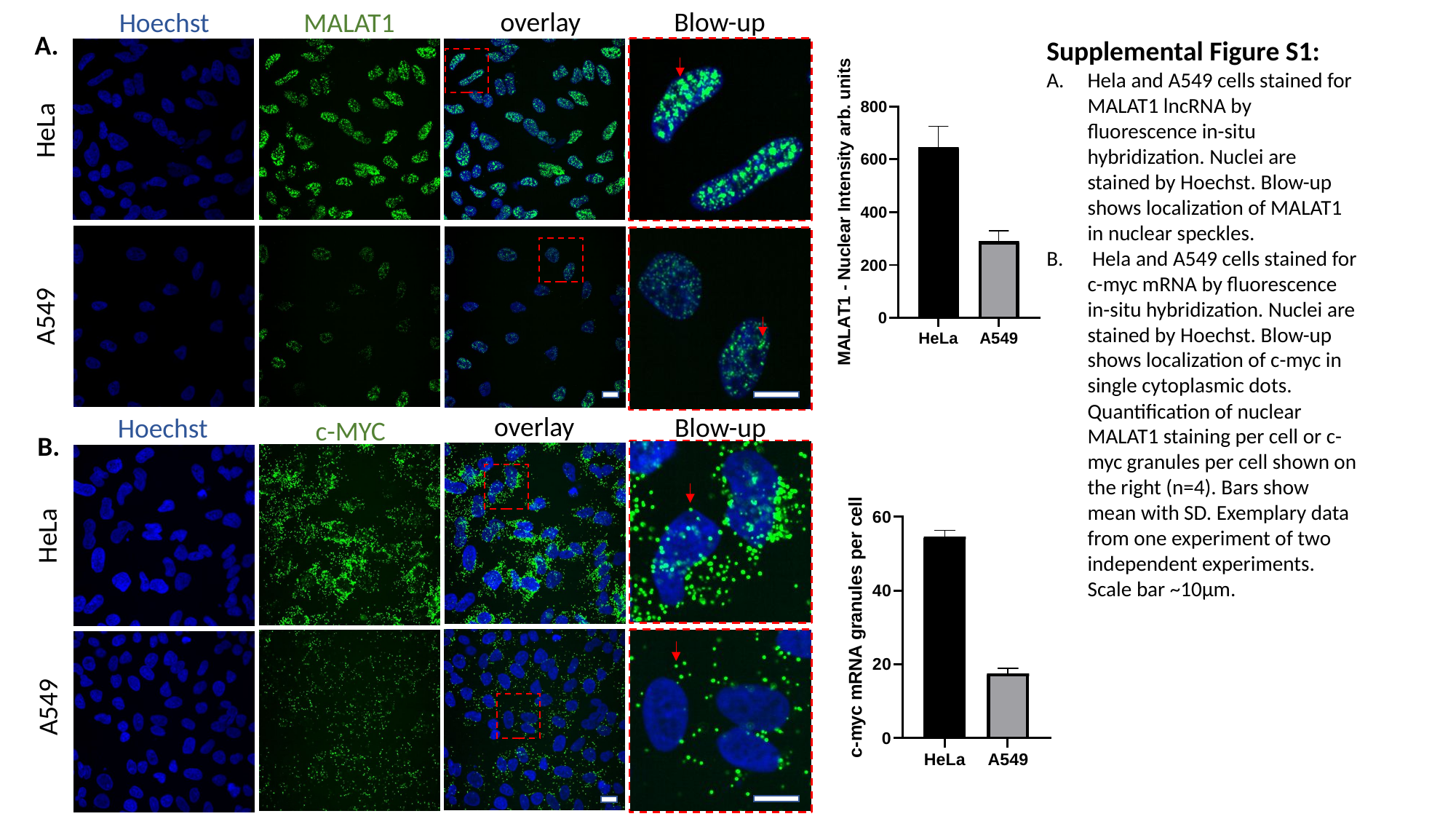

Blow-up
overlay
MALAT1
Hoechst
A.
Supplemental Figure S1:
Hela and A549 cells stained for MALAT1 lncRNA by fluorescence in-situ hybridization. Nuclei are stained by Hoechst. Blow-up shows localization of MALAT1 in nuclear speckles.
 Hela and A549 cells stained for c-myc mRNA by fluorescence in-situ hybridization. Nuclei are stained by Hoechst. Blow-up shows localization of c-myc in single cytoplasmic dots. Quantification of nuclear MALAT1 staining per cell or c-myc granules per cell shown on the right (n=4). Bars show mean with SD. Exemplary data from one experiment of two independent experiments. Scale bar ~10µm.
HeLa
A549
overlay
Blow-up
Hoechst
c-MYC
B.
HeLa
A549

## Slide 2
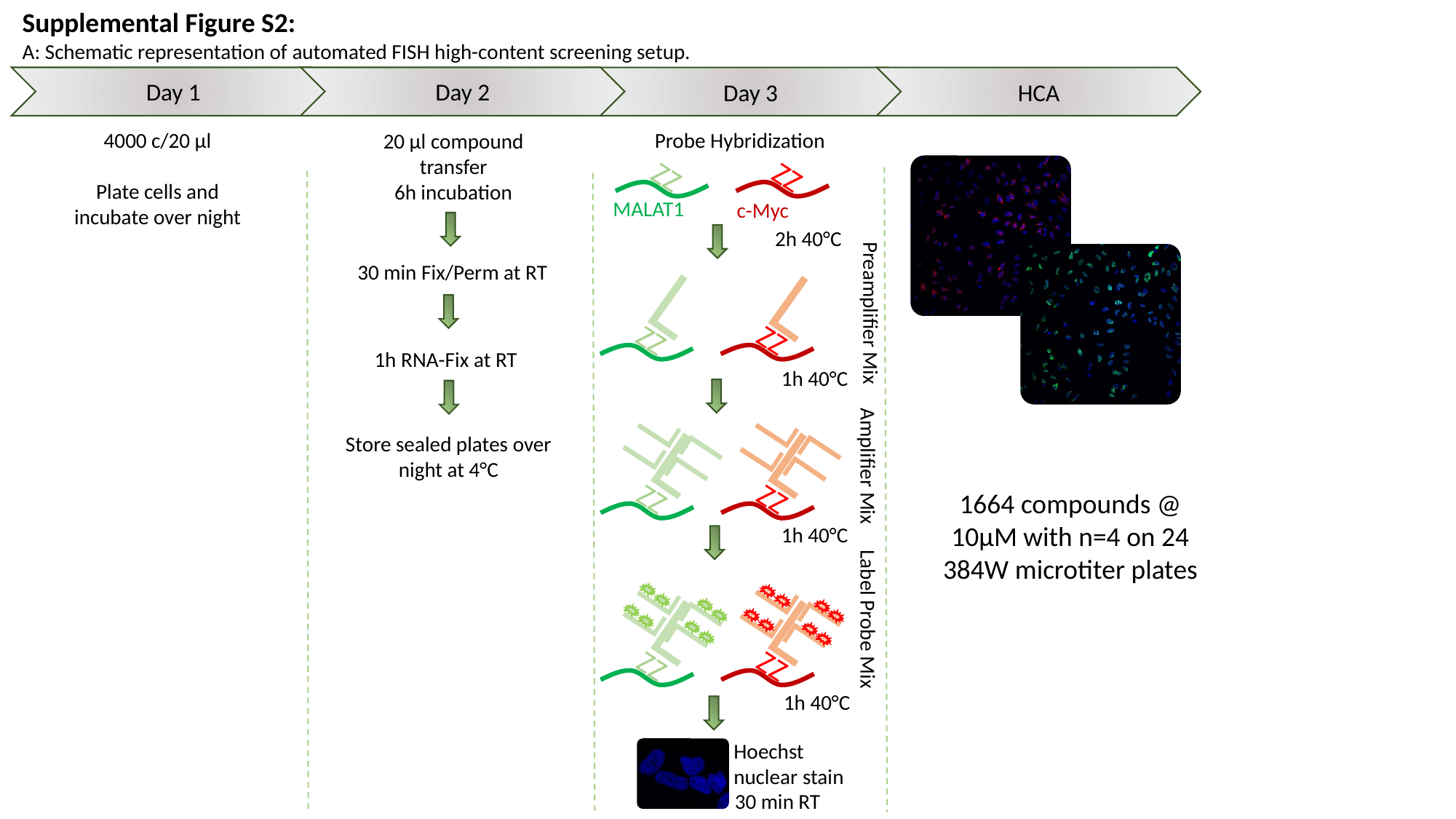

Supplemental Figure S2:
A: Schematic representation of automated FISH high-content screening setup.
Day 1
Day 2
Day 3
HCA
Probe Hybridization
4000 c/20 µl
Plate cells and incubate over night
20 µl compound transfer
6h incubation
ZZ
ZZ
MALAT1
c-Myc
2h 40°C
L
L
30 min Fix/Perm at RT
Preamplifier Mix
ZZ
ZZ
1h RNA-Fix at RT
1h 40°C
L
L
L
L
L
L
L
Store sealed plates over night at 4°C
L
L
L
ZZ
ZZ
1664 compounds @ 10µM with n=4 on 24 384W microtiter plates
Amplifier Mix
1h 40°C
L
L
L
L
L
L
L
L
L
L
Label Probe Mix
ZZ
ZZ
1h 40°C
Hoechst nuclear stain
30 min RT

## Slide 3
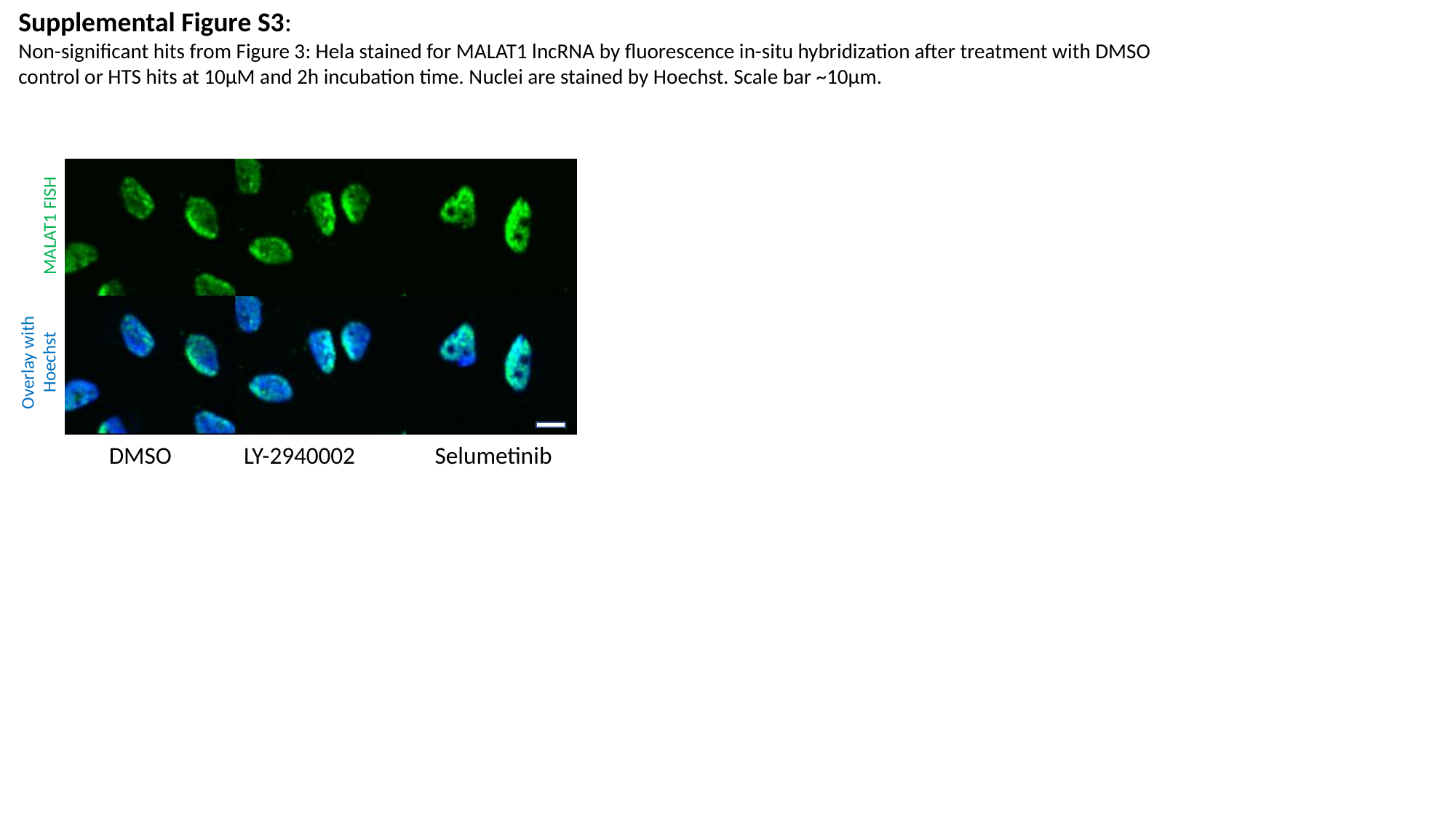

Supplemental Figure S3:
Non-significant hits from Figure 3: Hela stained for MALAT1 lncRNA by fluorescence in-situ hybridization after treatment with DMSO control or HTS hits at 10µM and 2h incubation time. Nuclei are stained by Hoechst. Scale bar ~10µm.
MALAT1 FISH
Overlay with Hoechst
DMSO
LY-2940002
Selumetinib

## Slide 4
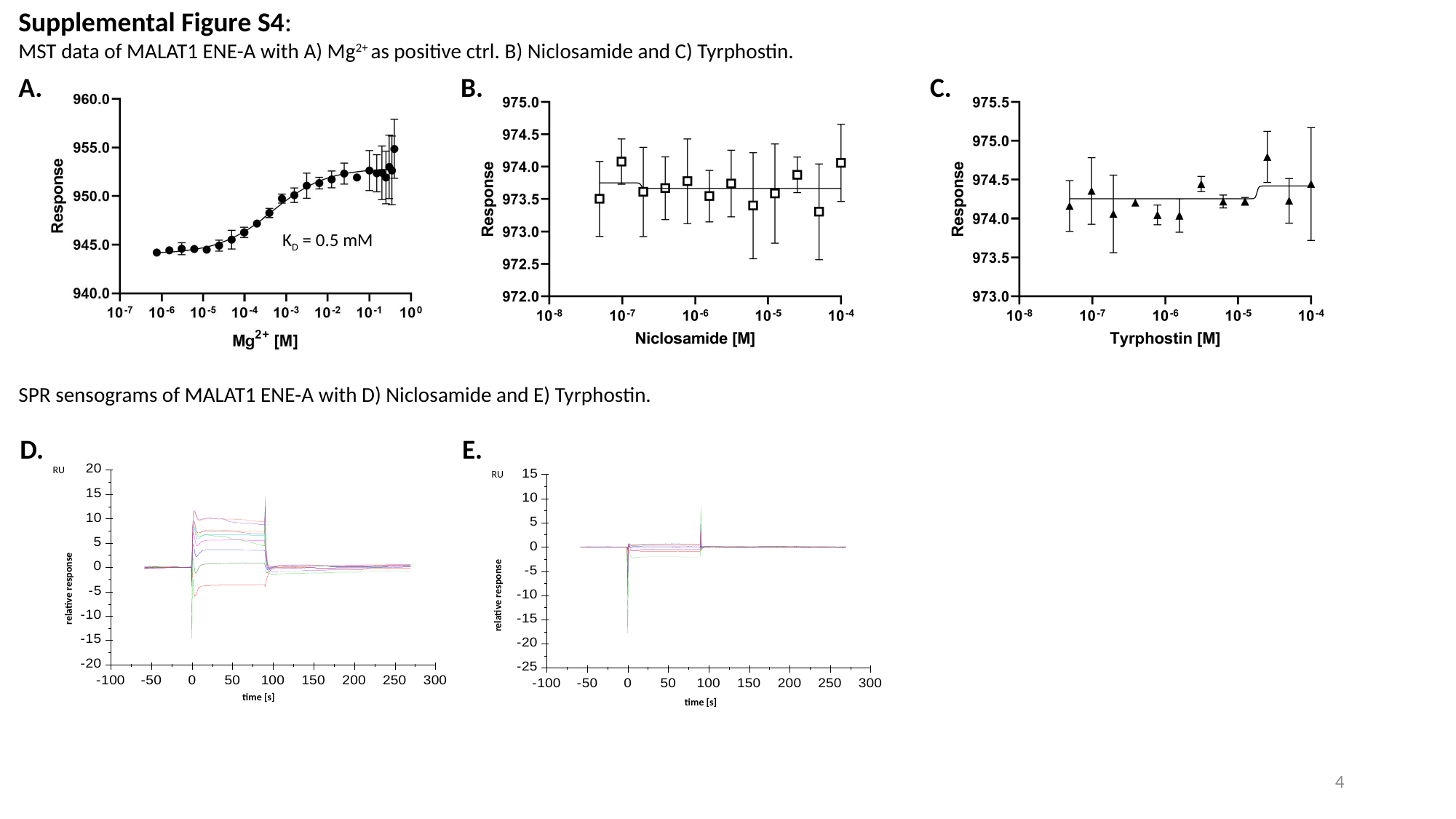

Supplemental Figure S4:
MST data of MALAT1 ENE-A with A) Mg2+ as positive ctrl. B) Niclosamide and C) Tyrphostin.
A.
B.
C.
KD = 0.5 mM
SPR sensograms of MALAT1 ENE-A with D) Niclosamide and E) Tyrphostin.
D.
E.
RU
relative response
time [s]
RU
relative response
time [s]
4

## Slide 5
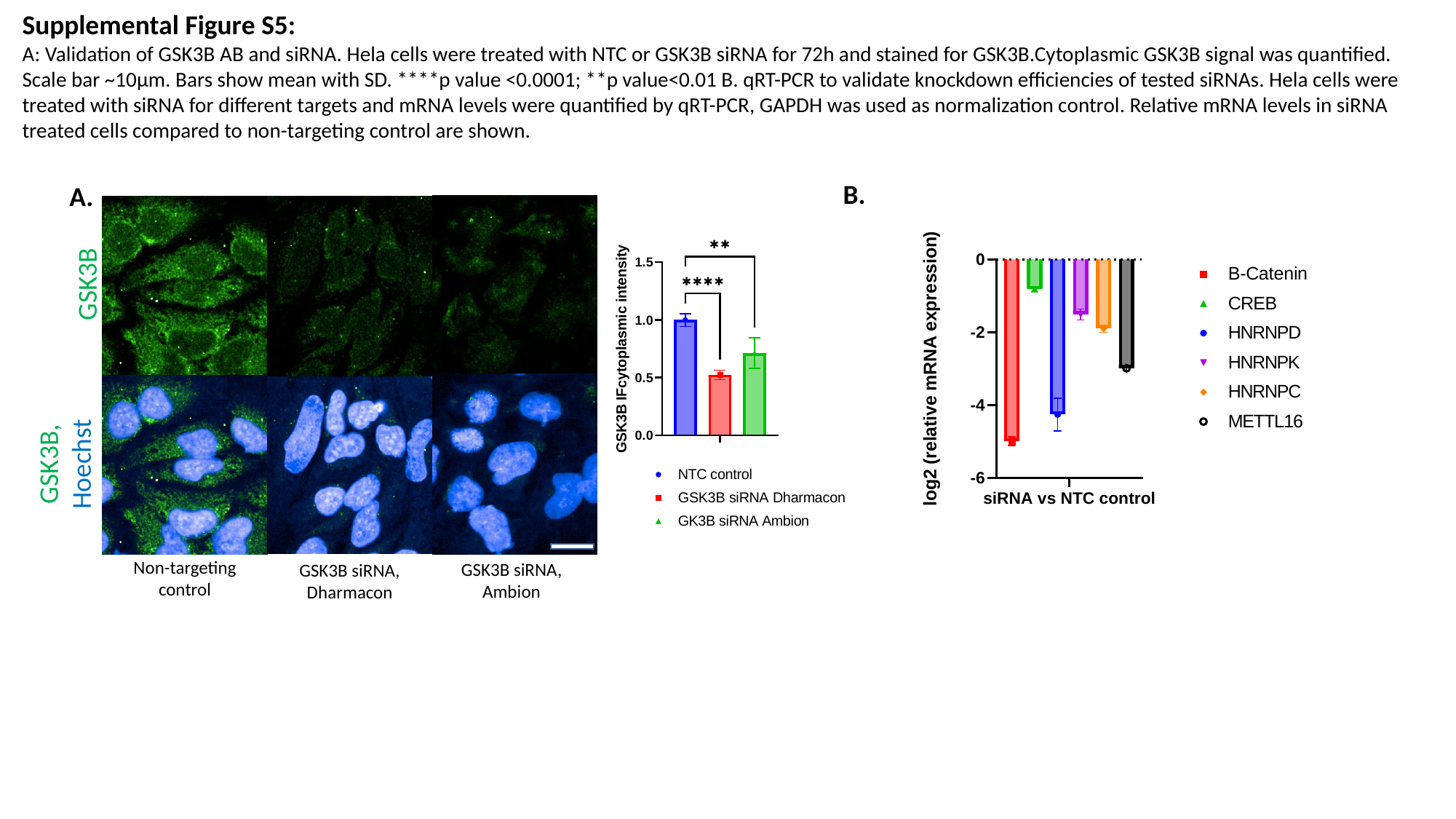

Supplemental Figure S5:
A: Validation of GSK3B AB and siRNA. Hela cells were treated with NTC or GSK3B siRNA for 72h and stained for GSK3B.Cytoplasmic GSK3B signal was quantified. Scale bar ~10µm. Bars show mean with SD. ****p value <0.0001; **p value<0.01 B. qRT-PCR to validate knockdown efficiencies of tested siRNAs. Hela cells were treated with siRNA for different targets and mRNA levels were quantified by qRT-PCR, GAPDH was used as normalization control. Relative mRNA levels in siRNA treated cells compared to non-targeting control are shown.
B.
A.
GSK3B
GSK3B, Hoechst
Non-targeting control
GSK3B siRNA, Ambion
GSK3B siRNA, Dharmacon

## Slide 6
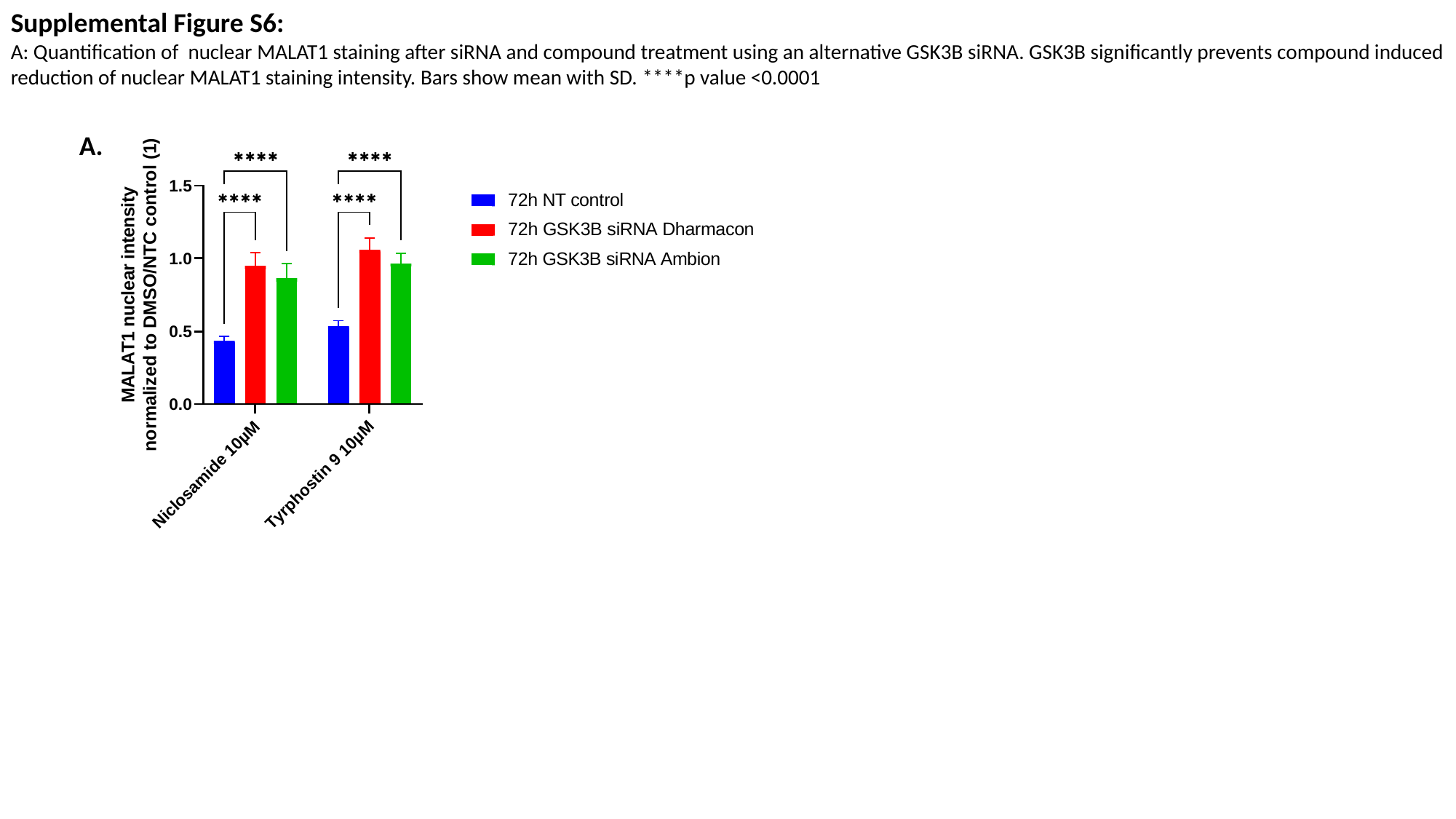

Supplemental Figure S6:
A: Quantification of nuclear MALAT1 staining after siRNA and compound treatment using an alternative GSK3B siRNA. GSK3B significantly prevents compound induced reduction of nuclear MALAT1 staining intensity. Bars show mean with SD. ****p value <0.0001
A.

## Slide 7
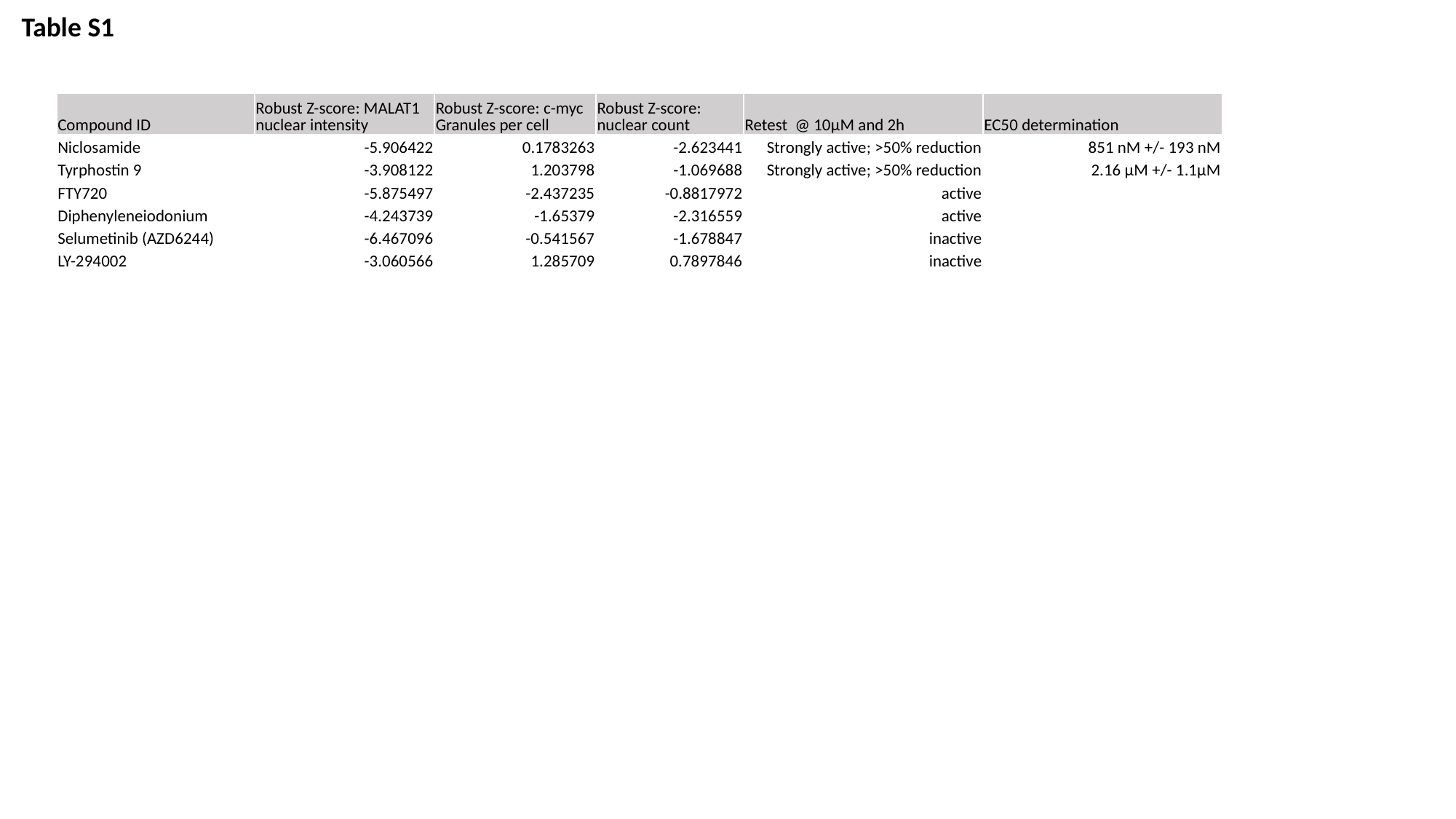

Table S1
| Compound ID | Robust Z-score: MALAT1 nuclear intensity | Robust Z-score: c-myc Granules per cell | Robust Z-score: nuclear count | Retest @ 10µM and 2h | EC50 determination |
| --- | --- | --- | --- | --- | --- |
| Niclosamide | -5.906422 | 0.1783263 | -2.623441 | Strongly active; >50% reduction | 851 nM +/- 193 nM |
| Tyrphostin 9 | -3.908122 | 1.203798 | -1.069688 | Strongly active; >50% reduction | 2.16 µM +/- 1.1µM |
| FTY720 | -5.875497 | -2.437235 | -0.8817972 | active | |
| Diphenyleneiodonium | -4.243739 | -1.65379 | -2.316559 | active | |
| Selumetinib (AZD6244) | -6.467096 | -0.541567 | -1.678847 | inactive | |
| LY-294002 | -3.060566 | 1.285709 | 0.7897846 | inactive | |

## Slide 8
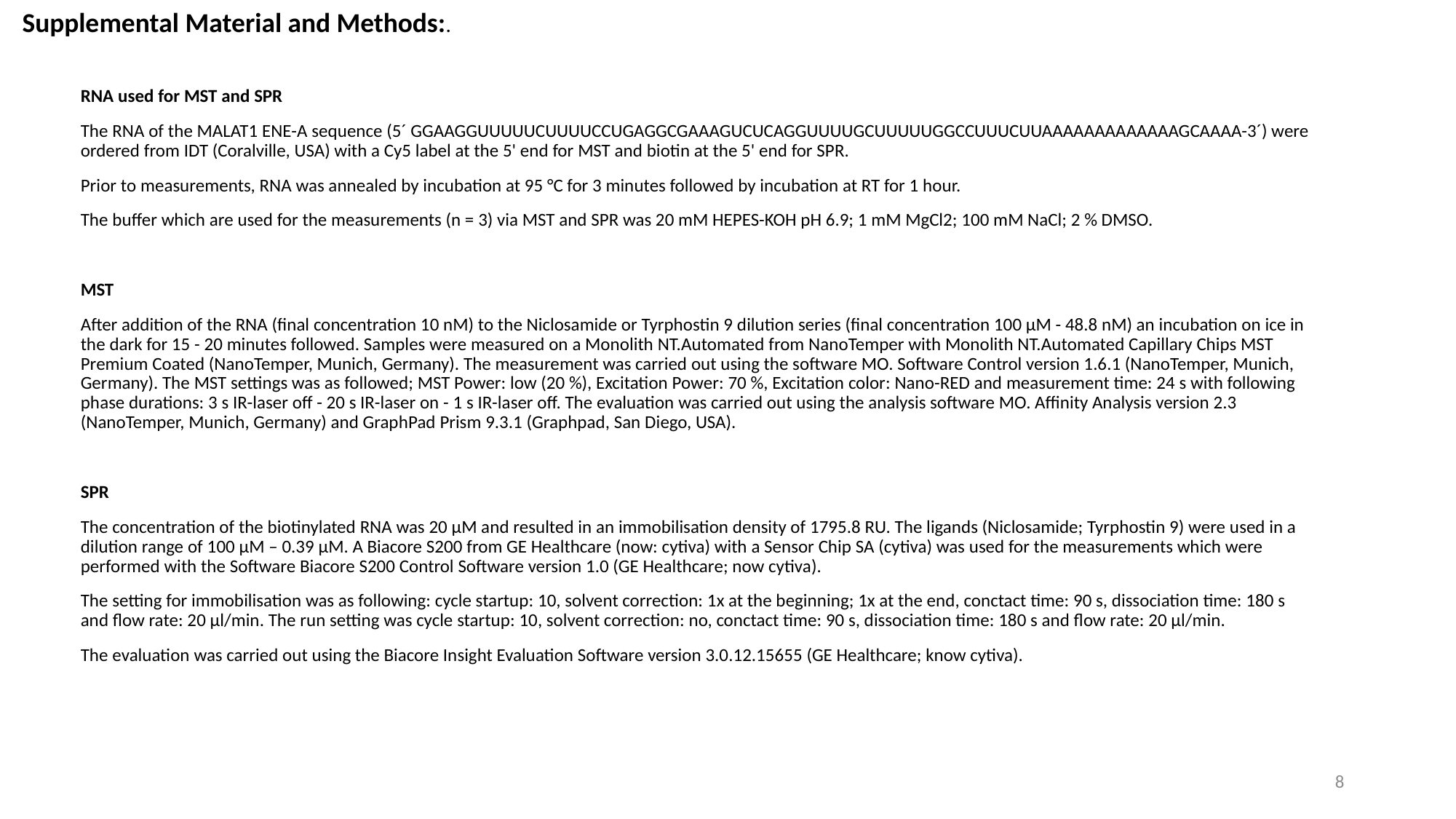

Supplemental Material and Methods:.
RNA used for MST and SPR
The RNA of the MALAT1 ENE-A sequence (5´ GGAAGGUUUUUCUUUUCCUGAGGCGAAAGUCUCAGGUUUUGCUUUUUGGCCUUUCUUAAAAAAAAAAAAAGCAAAA-3´) were ordered from IDT (Coralville, USA) with a Cy5 label at the 5' end for MST and biotin at the 5' end for SPR.
Prior to measurements, RNA was annealed by incubation at 95 °C for 3 minutes followed by incubation at RT for 1 hour.
The buffer which are used for the measurements (n = 3) via MST and SPR was 20 mM HEPES-KOH pH 6.9; 1 mM MgCl2; 100 mM NaCl; 2 % DMSO.
MST
After addition of the RNA (final concentration 10 nM) to the Niclosamide or Tyrphostin 9 dilution series (final concentration 100 μM - 48.8 nM) an incubation on ice in the dark for 15 - 20 minutes followed. Samples were measured on a Monolith NT.Automated from NanoTemper with Monolith NT.Automated Capillary Chips MST Premium Coated (NanoTemper, Munich, Germany). The measurement was carried out using the software MO. Software Control version 1.6.1 (NanoTemper, Munich, Germany). The MST settings was as followed; MST Power: low (20 %), Excitation Power: 70 %, Excitation color: Nano-RED and measurement time: 24 s with following phase durations: 3 s IR-laser off - 20 s IR-laser on - 1 s IR-laser off. The evaluation was carried out using the analysis software MO. Affinity Analysis version 2.3 (NanoTemper, Munich, Germany) and GraphPad Prism 9.3.1 (Graphpad, San Diego, USA).
SPR
The concentration of the biotinylated RNA was 20 µM and resulted in an immobilisation density of 1795.8 RU. The ligands (Niclosamide; Tyrphostin 9) were used in a dilution range of 100 µM – 0.39 µM. A Biacore S200 from GE Healthcare (now: cytiva) with a Sensor Chip SA (cytiva) was used for the measurements which were performed with the Software Biacore S200 Control Software version 1.0 (GE Healthcare; now cytiva).
The setting for immobilisation was as following: cycle startup: 10, solvent correction: 1x at the beginning; 1x at the end, conctact time: 90 s, dissociation time: 180 s and flow rate: 20 µl/min. The run setting was cycle startup: 10, solvent correction: no, conctact time: 90 s, dissociation time: 180 s and flow rate: 20 µl/min.
The evaluation was carried out using the Biacore Insight Evaluation Software version 3.0.12.15655 (GE Healthcare; know cytiva).
8
