# Supplementary material for: High Throughput FISH Screening Identifies Small Molecules That Modulate Oncogenic lncRNA MALAT1 via GSK3B and hnRNPs
Source: Noncoding RNA. 2023 Jan 3;9(1):2. doi: 10.3390/ncrna9010002 (PMC9844399; doi:10.3390/ncrna9010002)
Supplement: Supplementary file 1 [file ncrna-09-00002-s001.zip › 221207_Zablowsky_Supplement_MISFISHIE.pdf]

Description of FISH Experiments according to MISFISHIE:

Experiment design:

Assay type: *In situ* hybridization of A549 and Hela cells

Experiment design type: HTS, hit confirmation and mode of action studies using probes against c-myc, MALAT1 and NEAT1 RNAs.

Experimental factors: effect of small molecules different RNA species

Total number of assays performed in the experiment: except the primary HTS runs, at least three independent experiment repetitions were performed for each experiment.

URL of website:

ViewRNA Plus: ViewRNA™ Cell Plus Assay Kit (thermofisher.com)

ViewRNA Probe Set MALAT1: Lot # VA4-10912 (thermofisher.com)

ViewRNA Probe Set NEAT1: Product Details (thermofisher.com)

ViewRNA Probe Set c-myc: Product Details (thermofisher.com)

Contact information for the experiment: Patrick.Steigemann@gmail.com

Biomaterials:

Origin of specimens: A549 and Hela cell lines were obtained from tebu-bio or biomol. Both cell lines were cultured in RPMI1640 Medium (Gibco #21875-034 with 10% FBS (Biochrom #S0615) and 1% Penicillin-Streptomycin (Sigma Aldrich #P0781). Cells were maintained at 37 °C in a 5% CO_2 and 95% air incubator. For passaging and seeding cells were washed with DPBS (Gibco #14190-094) and trypsinized (TrypleE Gibco #12604-013).

Manner of preparation of the specimens and FISH staining: see Materials and Method section

FISH protocol and probes used:

ViewRNA Plus: ViewRNA™ Cell Plus Assay Kit (thermofisher.com)

ViewRNA Probe Set MALAT1: Lot # VA4-10912 (thermofisher.com)

ViewRNA Probe Set NEAT1: Product Details (thermofisher.com)

ViewRNA Probe Set c-myc: Product Details (thermofisher.com)

Imaging data and parameters:

Images were acquired using the automated confocal microscopy system Opera Phenix (Perkin Elmer, #HH14001000) and either 20x or 40x water objectives. Image analysis and quantification was carried out with the Harmony (PerkinElmer, Waltham, MA, USA) or MetaXpress software (Molecular

Devices) using custom-written scripts. Briefly, nuclei and cytoplasm were detected by Hoechst staining, the number of nuclei were counted per well and the intensity of the FISH signals was quantified as mean in the region of the nuclei and cytoplasm. For c-myc quantification c-myc granules were detected as granules per cell. Data was analyzed in Genedata Screener (Genedata, Basel, Switzerland) or Prism (Graphpad, San Diego, USA).
